# Role of an adenylyl cyclase isoform in ethanol's effect on cAMP regulated gene expression in NIH 3T3 cells

**DOI:** 10.1016/j.bbrep.2016.08.025

**Published:** 2016-09-03

**Authors:** Rebecca A. Hill, Wu Xu, Masami Yoshimura

**Affiliations:** Department of Comparative Biomedical Sciences, School of Veterinary Medicine, Louisiana State University, Baton Rouge, LA, USA

**Keywords:** Adenylyl cyclase, Ethanol, cAMP, CREB, TORC

## Abstract

Previous research has indicated that the cyclic AMP (cAMP) signal transduction system plays an important role in the predisposition to and development of ethanol abuse in humans. Our laboratory has demonstrated that ethanol is capable of enhancing adenylyl cyclase (AC) activity. This effect is AC isoform-specific; type 7 AC (AC7) is most enhanced by ethanol. Therefore, we hypothesized that the expression of a specific AC isoform will play a role on the effect of ethanol on cAMP regulated gene expression. We employed NIH 3T3 cells transfected with AC7 or AC3 as a model system. To evaluate ethanol's effects on cAMP regulated gene expression, a luciferase reporter gene driven by a cAMP inducing artificial promoter was utilized. Stimulation of AC activity leads to an increase in the reporter gene activity. This increase was enhanced in the presence of ethanol in cells expressing AC7, while cells expressing AC3 did not respond to ethanol. cAMP reporter gene expression was increased in the presence of 8-bromo-cAMP; this expression was not enhanced by ethanol. These observations are consistent with our hypothesis. The basal level of CREB phosphorylation was high and did not change by cAMP stimulation or in the presence of ethanol. However, there were significant changes in the TORC3 amount in nuclei depending on stimulation conditions. The results suggest that nuclear translocation of TORC3 plays a more important role than CREB phosphorylation in the observed changes in the cAMP driven reporter gene activity.

## Highlights

• Effect of ethanol on cAMP regulated gene expression is AC isoform dependent.
• cAMP regulated gene expression is most enhanced by ethanol in cells expressing AC7.
• Gene expression increases with pharmacologically relevant ethanol concentrations.
• TORC3 nuclear translocation is important for cAMP reporter gene activity.

## 1 Introduction

A second messenger molecule, cAMP, is capable of regulating many processes within an organism. These processes include: lipid and sugar metabolism, cell growth, cell differentiation, learning, and memory. Cyclic AMP is generated through the activation of a membrane-bound multi-protein system, whose components include adenylyl cyclase (AC), heterotrimeric G proteins, and G protein-coupled receptors (GPCR). The system is regulated by activation of GPCRs through various extracellular stimulants including: neurotransmitters, odorants, and hormones. Nine membrane-bound, mammalian AC isoforms, type 1 to type 9 (AC1-AC9), have been identified. Each AC isoform is uniquely regulated and is distinctly distributed within various tissues [1], [2], [3]. Changes in expression levels of AC have been reported for several pathological conditions; these changes in expression are AC isoform specific and have been found in brains of alcoholics [4], Alzheimer's disease patients [5], and heroin addicts [6]. Previous clinical research has implicated a link between cAMP signaling and ethanol abuse [7], [8], [9], [10], [11]. Cyclic AMP signaling elements including specific isoforms of AC: AC1, AC5, and AC8 have also been implicated to play a vital role in the behavioral and physiological responses to ethanol in animals [12], [13], [14].

Ethanol is capable of altering the activities of cAMP signaling pathways. This alteration is evident in animal models and model cell culture systems. Receptor-stimulated and/or stimulatory G protein (Gs)-activated AC activity is enhanced by acute ethanol exposure, while AC activity is decreased by chronic exposure [15]. ACs play an important role in cAMP-dependent activation of protein kinase A (PKA) as well as regulating cAMP response element binding protein (CREB) function. Studies indicate that CREB is a potential ethanol target and plays a central role in the process of ethanol addiction [16], [17], [18]. Levels of activated CREB, mediated by its Ser-133 phosphorylation, are altered during ethanol withdrawal [19]. CREB mediates the activation of cAMP-responsive genes by binding as a dimer to CRE; phosphorylated CREB (pCREB) then causes an increase in transcription. Changes in the activity of CREB and the expression of genes regulated by CREB have been associated with the abuse of drugs such as opiates, cocaine, and ethanol and are critical to drug addiction [18]. Recently, a new family of tissue and cell specific CREB co-activators has been discovered [20], [21]. These proteins, called transducer of regulated CREB activity (TORC), bind to the bZip domain of CREB. TORC1-3 are each sequestered in the cytoplasm and translocated into the nucleus by stimulating cAMP generation [22]. TORC1-3 also activate CRE-driven transcription; indicating that in addition to CREB, the TORC pathway may play a role in cAMP regulated gene expression. Furthermore, a new model of the active CREB complex necessary for expression of cAMP regulated genes has been proposed to have direct interaction of TORCs with pCREB and CBP/p300 [23].

We have previously shown that the activity of AC is enhanced by ethanol in an isoform-specific manner; AC7 is most responsive to ethanol, while AC3 is least responsive to alcohol [24]; AC7 activity can be significantly potentiated by pharmacologically relevant concentrations (less than 50 mM) of ethanol [25]. Humans can attain these concentrations in their blood by consuming alcoholic beverages. The current study examines the effects of ethanol on cAMP regulated reporter gene expression. We hypothesize that AC is one of the main targets of ethanol's action within the cAMP-signaling system; ethanol's effect on cAMP signaling will be affected by the AC isoform expressed within the cells. Thus, an alteration in cAMP inducible gene expression during exposure to ethanol will depend on AC isoform expressed in the cells. The objective of this study is to examine whether ethanol's effect on the expression cAMP inducible gene is dependent on the AC isoform expressed in cells. Two AC isoforms, AC3 and AC7, were specifically selected for this study because of their contrasting responses to ethanol stimulation. NIH 3T3 cells have been utilized in numerous cAMP regulated gene expression studies. It is well established that NIH 3T3 cells respond to reagents which increase cAMP and increase cAMP inducible gene expression [26]. Thus, this is an ideal cell line for the current study. The dopamine D_1A_ receptor (DRD1A) is a Gs coupled receptor; stimulation with dopamine will activate the cAMP signaling pathway. The NIH 3T3 cells in our laboratory do not respond to dopamine without exogenous dopamine D_1A_ receptor expression. Thus, dopamine treatment will only activate the cAMP signaling pathway in NIH 3T3 cells that are transfected with DRD1A. Cyclic AMP levels in NIH 3T3 cells were monitored by Epac1-camp, a fluorescent resonance energy transfer (FRET) based cAMP sensor, and a radioisotope based cAMP accumulation. Cyclic AMP regulated gene expression was examined using a firefly luciferase reporter gene linked to GAL4 DNA and GAL4-CREB fusion protein. The changes in TORC nuclear translocation and the phosphorylation of CREB were investigated via western blotting. The results provide valuable insight into the mechanisms through which ethanol affects cAMP inducible gene expression.

## 2 Materials and methods

### 2.1 Reagents

Dopamine (DA), 8-bromo-cAMP, and ethanol were purchased from Sigma-Aldrich (St. Louis, MO). Prostaglandin E_1_ (PGE_1_) was purchased from Cayman Chemical (Ann Arbor, MI).

### 2.2 Plasmid DNA

Plasmids encoding AC3, AC7 [27], Epac1-camps [28], GAL4-CREB [29], firefly luciferase containing 5xGAL4 binding sites [30], SV40-Renilla reporter (Promega, Madison, WI), D_1A_ dopamine receptor (DRD1A) [31], and green fluorescent protein (GFP) (Clontech, Mountain View, CA) were utilized.

### 2.3 Cell culture and transfection

NIH 3T3 cells were maintained in 30 ml MEM with 10% fetal bovine serum (FBS), penicillin (50 μg/ml), streptomycin (50 μg/ml), and neomycin (100 μg/ml) in plastic flasks (225 cm^2^) at 37 °C in a humidified atmosphere of 95% air and 5% CO_2_. Cells were transiently transfected using Calfectin (SignaGen, Rockville, MD) according to manufacturer instructions. GFP or Epac1-cAMP expressed in cells was observed using an epifluorescence microscope in order to monitor transfection efficiency.

### 2.4 Fluorescence imaging of live cells

Transfected NIH 3T3 cells were added to the coverslip coated with Poly(ethyleneimine) (Sigma Aldrich, St. Louis, MO) 24 h prior to the experiment. The coverslip was assembled in a perfusion chamber (~10 ml/min flow rate and 87 µl/mm depth) and then attached to a perfusion valve control system (Warner Instruments, Hamden, CT). The fluorescent imaging workstation, filter sets, and buffer composition was described previously [32].

### 2.5 FRET measurements

The method of sensitized FRET measurement was previously described [33]. Corrected FRET (cFRET) and normalized FRET (nFRET) were calculated as previously described [32]. nFRET values were normalized by dividing by the mean nFRET value of the first 18 time points.

### 2.6 Luciferase assay

24 h post-transfection, cells were transferred to 24-well culture plates. Before lysate preparation, cells were treated with DA, PGE_1_, or 8-bromo-cAMP in the presence or absence of ethanol. Firefly and Renilla luciferase activities were analyzed using a Dual Luciferase Assay System (Promega, Madison, WI) and a Synergy HT plate reader (BioTek Instruments, Winooski, VT) according to manufacturer instructions. Firefly luciferase activities were normalized against Renilla luciferase activities. The concentrations of DA, PGE_1_, 8-bromo-cAMP, and ethanol were chosen in order to obtain maximum effects based on predetermined concentration dependent curves.

### 2.7 Nuclear extract preparation and western blotting

Following stimulation, cells were washed with cold phosphate-buffered saline (PBS) and collected via scraping. Nuclear extracts were prepared using a NE-PER kit from Pierce (Rockford, IL). Protein concentration in the extracts was determined using the BCA protein assay kit (Pierce, Rockford, IL). The extracts were stored at −80 °C prior to use. Proteins (10 µg per lane) were separated by 12.5% SDS-PAGE and transferred to a nitrocellulose membrane. Western blotting was carried out using following primary antibodies according to manufacturer protocol: anti-CREB (06–863, Millipore, Billerica, MA), *anti*-pCREB (06–519, Millipore, Billerica, MA), anti-TORC1 (4E7-C1-F9-E6, GeneTex, Irvine, CA), anti-TORC2 (ab167129, Abcam, Cambridge, MA), anti-TORC3 (EPR3440, GeneTex, Irvine, CA), and anti-β-actin (#4967, Cell Signaling, Danvers, MA). Appropriate secondary antibodies conjugated with horseradish peroxidase were obtained from Jackson ImmunoResearch (West Grove, PA). Signals were visualized by chemiluminescence using ECL detection reagents (GE Healthcare, Piscataway, NJ), captured by ChemiDoc Touch Imaging System (Bio-Rad, Hercules, CA), quantified using ImageJ software [34], and normalized to β-actin for comparison.

### 2.8 Statistics

Values are expressed as mean ± standard error of mean (SEM). Statistical significance of the differences in values was examined by Student's *t*-test, one-way ANOVA, two-way ANOVA, or two-way repeated-measures ANOVA, which is specified in figure legends. Holm–Sidak method was employed for pairwise comparison after ANOVA. Differences of values were considered statistically significant when the p value was less than 0.05. Analyses were carried out using SigmaStat (SyStat Software, San Jose, CA).

## 3 Results and discussion

### 3.1 Effect of ethanol on cAMP in NIH 3T3 cells is AC isoform dependent

**Fig. 1:**
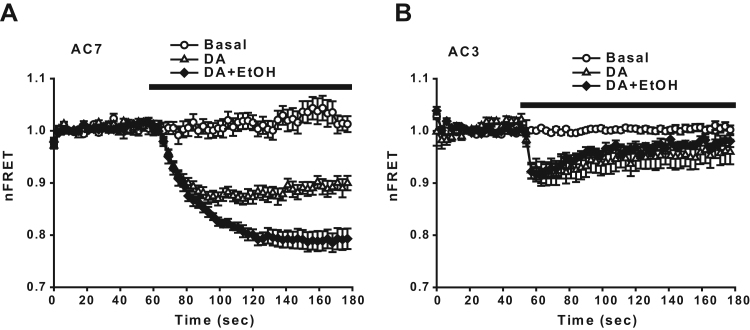
(A) FRET-based real-time monitoring of cAMP in cells expressing AC7 and DRD1A (Basal, n=8; DA, n=10; DA+Ethanol, n=8). Normalized FRET (nFRET) values are plotted over time. The bar on top of the graph indicates 2 min duration of 3 µM DA±200 mM ethanol (EtOH) treatment; all treatments are significantly different from one another at all time points past 100 s (p<0.05, two-way repeated-measures ANOVA). (B) FRET-based real-time monitoring of cAMP in cells expressing AC3 and DRD1A (Basal, n=13; DA, n=11; DA+EtOH, n=9). Cell treatment and statistical analysis were similar to (A). Basal is significantly different from DA or DA+EtOH between 60 and 180 s, there is no significant difference between DA and DA+EtOH.

Real-time changes in cAMP in NIH 3T3 cells expressing either AC7 or AC3 was monitored by co-expressing Epac1-camps and DRD1A and stimulating with DA±ethanol for 2 min. As an indicator of changes in cAMP, normalized nFRET values were plotted over time (Fig. 1). nFRET quickly decreased after the addition of DA in cells expressing AC7 (Fig. 1A); this decrease indicates an increase in intracellular cAMP. After perfusion with DA, the value of nFRET reached its minimum within 20 s and remained constant during the remainder of the treatment; when DA + ethanol were added, nFRET was further decreased, which indicated higher level of cAMP in cells compared to the cells treated with DA alone. During DA stimulation, cAMP levels of cells expressing AC3 quickly increased then slowly decreased (Fig. 1B). Addition of ethanol did not change the time course or amount of cAMP in cells expressing AC3 compared to addition of DA alone. Cyclic AMP accumulation assay also showed similar ethanol effects (Supplementary Fig. S1). These results confirm our previous observations in Hela and HEK 293 cells [24], [32], suggesting that the isoform specific effect of ethanol on AC activity can be observed in many different mammalian cell types and that AC is the main target of ethanol effect on cAMP amount in cells.

### 3.2 Effect of DA and ethanol on cAMP regulated reporter gene expression

**Fig. 2:**
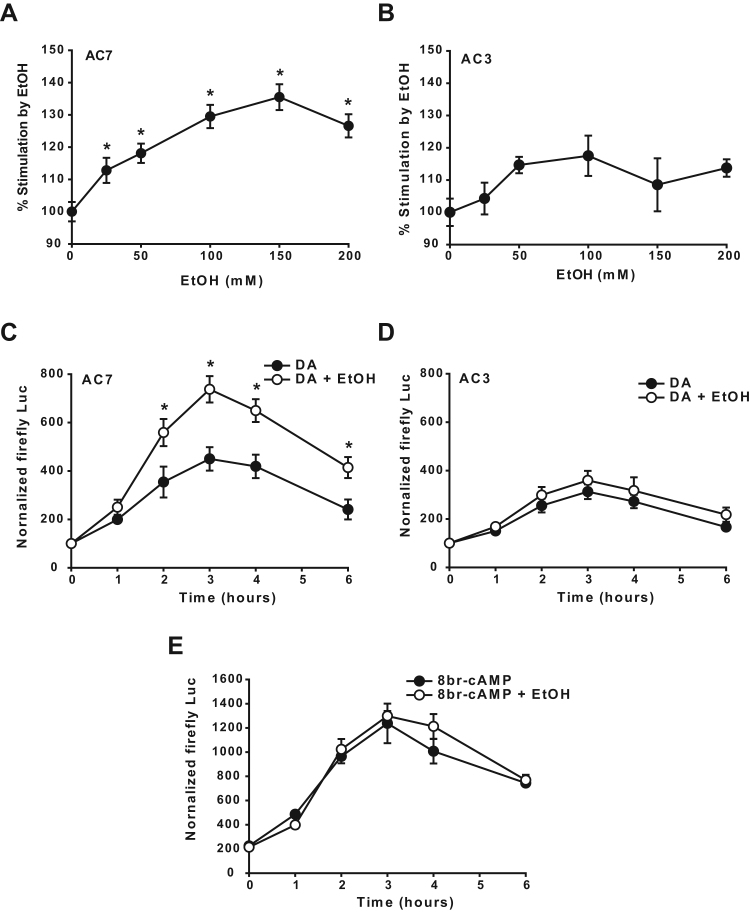
(A) Effect of ethanol on firefly luciferase activity. Cells expressing DRD1A and AC7 were incubated with 3 µM DA in the presence of 0, 25, 50, 100, 150, 200 mM ethanol (EtOH) for 3 h. Percent stimulation by ethanol is plotted (n =9 to 12). *Value is significantly higher than DA alone (p<0.05, one-way ANOVA). (B) Effect of ethanol on firefly luciferase activity. Cells expressing DRD1A and AC3 were treated and analyzed similar to (A) (n=3). (C) Time course of reporter gene activity stimulated by DA and ethanol in cells expressing DRD1A and AC7. Cells were incubated with 3 µM DA±150 mM ethanol for 0, 1, 2, 3, 4, or 6 h (n=6). *Value is significantly higher in the presence of ethanol (p<0.05, two-way ANOVA). (D) Time course of reporter gene activity stimulated by DA and ethanol in cells expressing DRD1A and AC3 (n=6). (E) Time course of reporter gene activity stimulated by 8br-cAMP and ethanol. Cells transfected with AC7 were incubated with 1 mM 8br-cAMP ±150 mM ethanol for 0, 1, 2, 3, 4, or 6 h as indicated (n=3).

DA caused a concentration dependent increase in firefly luciferase activity in cells expressing AC7 and DRD1A (Supplementary Fig. S2). Maximum firefly luciferase activity occurred at concentrations greater than 0.3 µM DA. Cells expressing DRD1A and either AC7 or AC3 were incubated with DA and varying ethanol concentrations for 3 h. Firefly luciferase activity was significantly increased by ethanol at all concentrations including 25 mM in AC7 expressing cells and was maximized at 150 mM ethanol (Fig. 2A). No significant enhancement by the addition of ethanol was evident in AC3 expressing cells (p>0.05; Fig. 2B). The results are also consistent with the isoform specific effect of ethanol on AC activity [24]. Cells expressing DRD1A and either AC7 or AC3 were incubated with DA ± ethanol (150 mM) for varying durations up to 6 h. Firefly luciferase activity reached its maximum after 3 h of incubation with DA then decreased for the duration of the experiment. In cells expressing AC7, a significant increase (p<0.05) in cAMP regulated reporter gene expression was detected at all time points past 2 h by the addition of DA+ethanol when compared to the values observed with DA alone (Fig. 2C). In cells expressing AC3, cAMP regulated firefly luciferase activity was not significantly enhanced by DA+ethanol over values obtained with DA alone (p>0.05; Fig. 2D). 8-bromo-cAMP was utilized to uncouple AC from the cAMP signaling pathway. 8-bromo-cAMP concentration curve showed a similar pattern to that of DA, with maximized firefly luciferase activity occurring at concentrations greater than 1 mM (data not shown). Cells expressing DRD1A and AC7 were stimulated with DA ± ethanol or 8-bromo-cAMP ± ethanol for 3 h. In cells stimulated with DA + ethanol, a significant increase in cAMP regulated reporter gene expression was detected over that observed with DA alone. However, there was no significant change between reporter gene activity in cells stimulated with 8-bromo-cAMP alone compared to cells stimulated with 8-bromo-cAMP + ethanol (Supplementary Fig. S3). In the presence of 8-bromo-cAMP, firefly luciferase activity increased during the first 3 h and then decreased. This is similar to the time course observed in the presence of DA. Firefly luciferase activity was not increased by 8-bromo-cAMP + ethanol over values obtained with 8-bromo-cAMP alone during the 6 h incubation period (Fig. 2E). These results indicate that the effect of ethanol on cAMP regulated reporter gene expression is due to the enhancement of AC activity by ethanol, resulting in a higher level of cAMP. PGE_1_ (10 µM) was used to stimulate cAMP generation without an exogenously expressed GPCR. Cells expressing AC7 were stimulated with PGE_1_, ethanol, or PGE_1_+ ethanol for 3 h. In cells stimulated with PGE_1_+ ethanol, a significant increase in cAMP regulated reporter gene expression was detected compared to those observed with basal, PGE_1_, or ethanol alone (Supplementary Fig. S4). These results indicate that enhancement of cAMP regulated reporter gene expression by ethanol can be observed by stimulating endogenous GPCRs in cells expressing AC7.

### 3.3 Effect of ethanol on CREB phosphorylation and TORC nuclear translocation

**Fig. 3:**
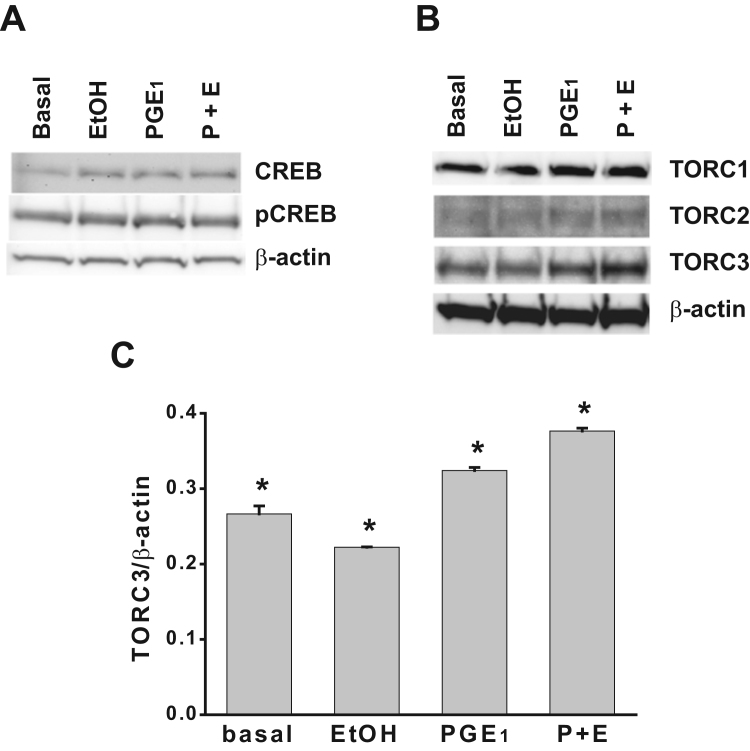
(A) Immunodetection of pCREB. Western blot analysis of nuclear extracts from NIH 3T3 cells incubated in the presence or absence of 10 µM PGE_1_ and 150 mM ethanol (EtOH) for 30 min (B) Immunodetection of TORCs. Western blot analysis of nuclear extracts from NIH 3T3 cells incubated in the presence or absence of 10 µM PGE_1_ and 150 mM ethanol for 30 min (C) Changes in TORC3 was normalized using β-actin (n=3). All pair-wise comparisons are significant (p<0.05, one-way ANOVA).

Initially, we tried to detect the GAL4-CREB fusion protein that is directly responsible for the observed firefly luciferase expression. This protein has the same epitopes as the endogenous CREB protein but has a different molecular weight. We could not detect this protein in the nuclear extract prepared from the NIH 3T3 cells transfected with the plasmid encoding this protein (data not shown). Thus, we examined the endogenous CREB as a surrogate since we assume that the regulation of the GAL4-CREB by cAMP signaling is very similar to that of the endogenous CREB. Studies show that the level of pCREB increases when cAMP generation is stimulated [35], [36], [37]. Incubation with ethanol increased the level of pCREB in NG108–15 cells [38] and NIH 3T3 cells [39]. In the current study the level of pCREB in nuclear extracts from NIH 3T3 cells transfected with AC7 was not significantly increased by stimulation with PGE_1_, ethanol, or PGE_1_+ ethanol (Fig. 3A and Supplementary Fig. S5). Levels of CREB and pCREB were examined at varying time points; no differences among the four conditions were detected (data not shown). The notable difference between current study and previous studies is that the NIH 3T3 cells in our laboratory appear to have a much higher basal pCREB level than previously reported; the level of pCREB appears to be independent of the stimulation conditions. Culture conditions in the current study may have caused a high basal level of pCREB or our NIH 3T3 cells may have unknown alterations compared to cells in previous studies. In either case, the changes in the level of pCREB did not correlate with the changes in expression level of the cAMP regulated reporter gene. The results suggest that the phosphorylation of GAL4-CREB is also high and does not respond to the different stimulations.

We then examined the levels of TORC1-3 in the nuclear extracts of NIH 3T3 cells transfected with AC7. TORCs translocate from the cytoplasm to the nucleus by stimulation of cAMP signaling and participate in the activation of CRE-driven transcription [22]. Western blotting of TORC1-3 detected signals of expected size, however, TORC2 signals were too weak to quantify (Fig. 3B). TORC1 signals did not significantly change with different stimulation conditions (p>0.05; Fig. 3B and Supplementary Fig. S6). TORC3 signals showed that PGE_1_ increases TORC3 translocation to the nucleus; ethanol further enhances this translocation (Fig. 3B and C). The results suggest that translocation of TORC3 reflects stimulation of AC7 by PGE_1_ and ethanol, while that of TORC1 is not depending on cAMP signaling. The difference in response of TORC1 and TORC3 to the stimulation conditions could be due to several factors. It could be due to a difference in sensitivity of TORC1 and TORC3 to the translocation signal, or there could be a difference in sub-cellular localization of TORC1 and TORC3 and their accessibility to the translocation signal. Alternatively, this could be due to different populations of cells within our NIH 3T3 cells which differentially express TORC1, TORC3 and factors involving their nuclear translocation. Among the protein factors examined, changes of TORC3 appear to correlate best with the observed changes in the expression level of the cAMP regulated reporter gene.

In conclusion, our findings indicate that the observed ethanol effect on cAMP regulated reporter gene expression is caused by a change in the amount of cAMP present in the cells, which is dictated by the isoform of AC expressed in the cells. The observed ethanol effect is AC isoform-specific and ethanol concentration-dependent. These results show that ethanol's effect on AC activity plays a major role on the expression of a cAMP regulated reporter gene. The increase in cAMP by addition of ethanol in cells expressing AC7 is correlated with the increase in reporter gene activity and the level of TORC3 in the nucleus. Further research is needed to elucidate the role of specific AC isoform in the effect of ethanol on cAMP regulated gene expression *in vivo*.
