# Supplementary material for: Role of an adenylyl cyclase isoform in ethanol's effect on cAMP regulated gene expression in NIH 3T3 cells
Source: Biochem Biophys Rep. 2016 Sep 3;8:162–7. doi: 10.1016/j.bbrep.2016.08.025 (PMC5467537; doi:10.1016/j.bbrep.2016.08.025)
Supplement: Supplementary file 3 — Supplementary material [file mmc4.pdf]

Role of an adenylyl cyclase isoform in ethanol's effect on cAMP regulated gene expression in NIH 3T3 cells

Rebecca A. Hill, Wu Xu, and Masami Yoshimura

Supplementary Information

Materials and Methods

cAMP Accumulation Assay

Amounts of cAMP in the transfected NIH 3T3 cells were assessed by the cAMP accumulation assay as described previously [1]. Briefly, the intracellular ATP pool was labeled with 3.0 μCi/ml of [2,8-^{3}H]adenine. Cells were treated with 10 μM DA ± 200 mM EtOH for 1 minute at 37°C. The reaction was terminated by adding 50 μl of 100% (w/v) trichloroacetic acid. ATP and cAMP contents of each well were separated through Dowex 50 and neutral alumina columns as described previously [2] and quantified by liquid scintillation spectrometer. [α-^{32}P]ATP and [8-^{14}C]cAMP were added as internal standards in order to monitor recovery of ATP and cAMP through column chromatography.

Reference

[1] M. Hasanuzzaman, M. Yoshimura, Effects of straight chain alcohols on specific isoforms of adenylyl cyclase. Alcohol Clin.Exp.Res. 34 (2010) 743-749.

[2] Y. Salomon, C. Londos, M. Rodbell, A highly sensitive adenylate cyclase assay. Anal.Biochem. 58 (1974) 541-548.

Supplementary Fig. S1

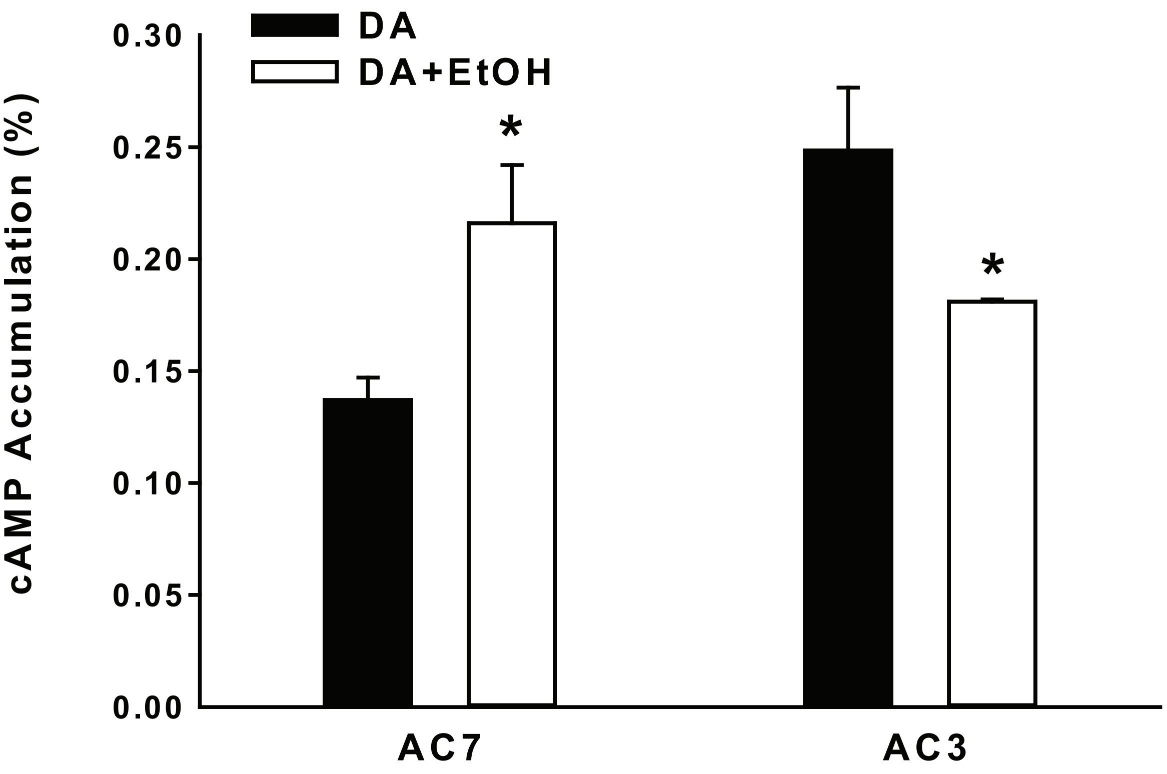

Supplementary Fig. S1. Effects of DA and EtOH on cAMP in cells expressing DRD1A and AC7 or AC3. cAMP accumulation assays were carried out in the presence of 10 μM DA ± 200 mM EtOH for 1 minute. Percent increase in cAMP accumulation by addition of DA ± EtOH is displayed (n =3). *Values significantly differ from those of DA alone ($p < 0.05$, student t-test).

Supplementary Fig. S2

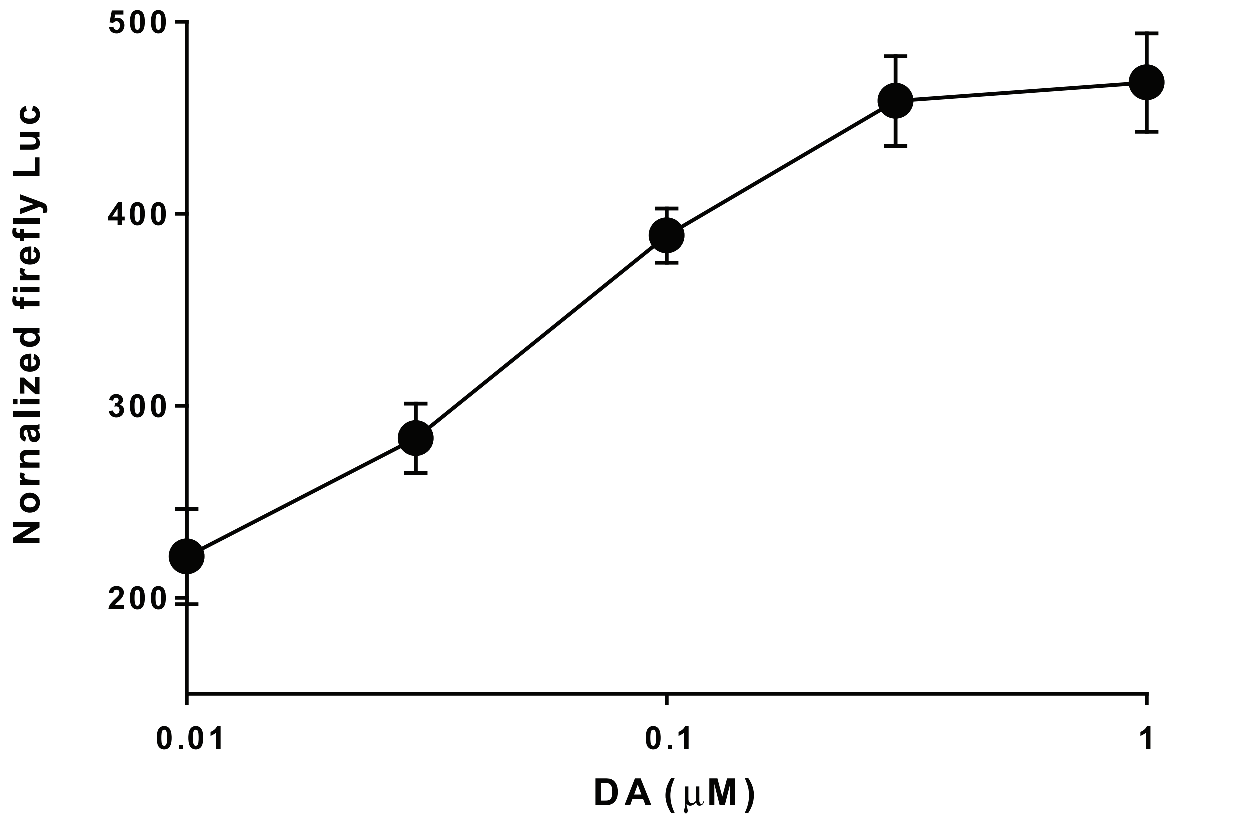

Supplementary Fig. S2. Effects of DA on cAMP stimulated reporter gene expression in cells expressing DRD1A and AC7. Cells were incubated in the presence of 0, 0.01, 0.03, 0.1, 0.3, 1 μM DA for 3 hours (n = 3). Basal activity in the absence of DA was 209 ± 15.1.

Supplementary Fig. S3

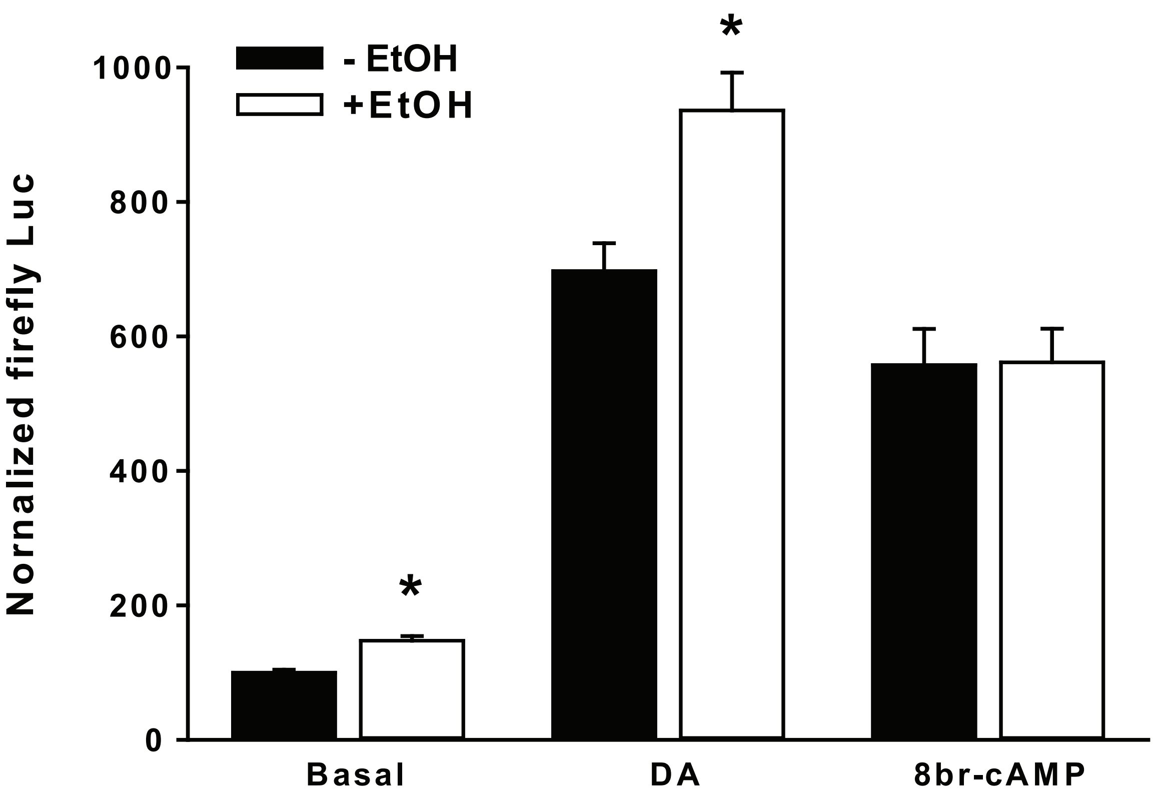

Supplementary Fig. S3. Effects of 8-bromo-cAMP on reporter gene activity in cells expressing DRD1A and AC7. Cells were incubated with 3 μM DA or 1 mM 8-bromo-cAMP ± 150 mM EtOH for 3 hours (n = 12). Basal received solvent only. *Value is significantly higher in the presence of EtOH ($p < 0.05$, student t-test).

Supplementary Fig. S4

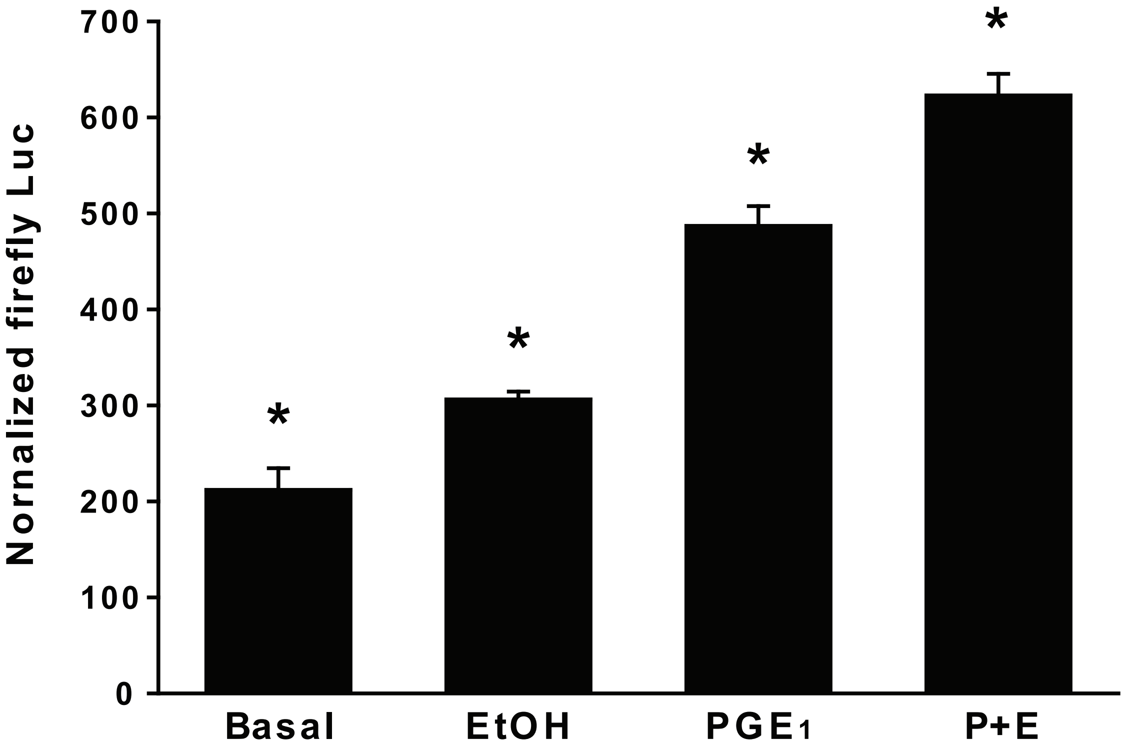

Supplementary Fig. S4. Effects of PGE_1 and EtOH on cAMP stimulated reporter gene expression in cells expressing AC7. Cells were incubated with 10 μM PGE_1 and 150 mM EtOH for 3 hours (n = 3). Basal received solvent only. All pair-wise comparisons are significant ($p < 0.05$, one-way ANOVA).

Supplementary Fig. S5

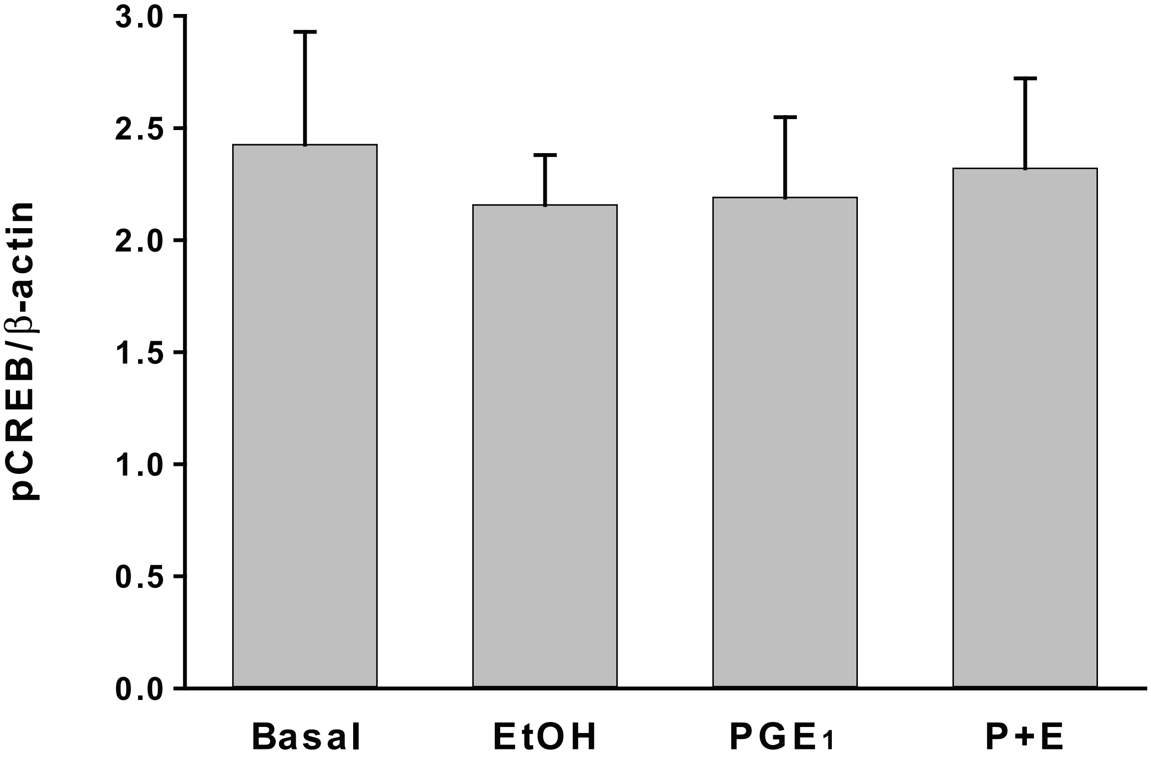

Supplementary Fig. S5. Immunodetection of pCREB. Changes in pCREB were normalized using β-actin (n = 3). Changes in pCREB in response to cAMP pathway activation or EtOH treatment are not significant (one-way ANOVA).

Supplementary Fig. S6

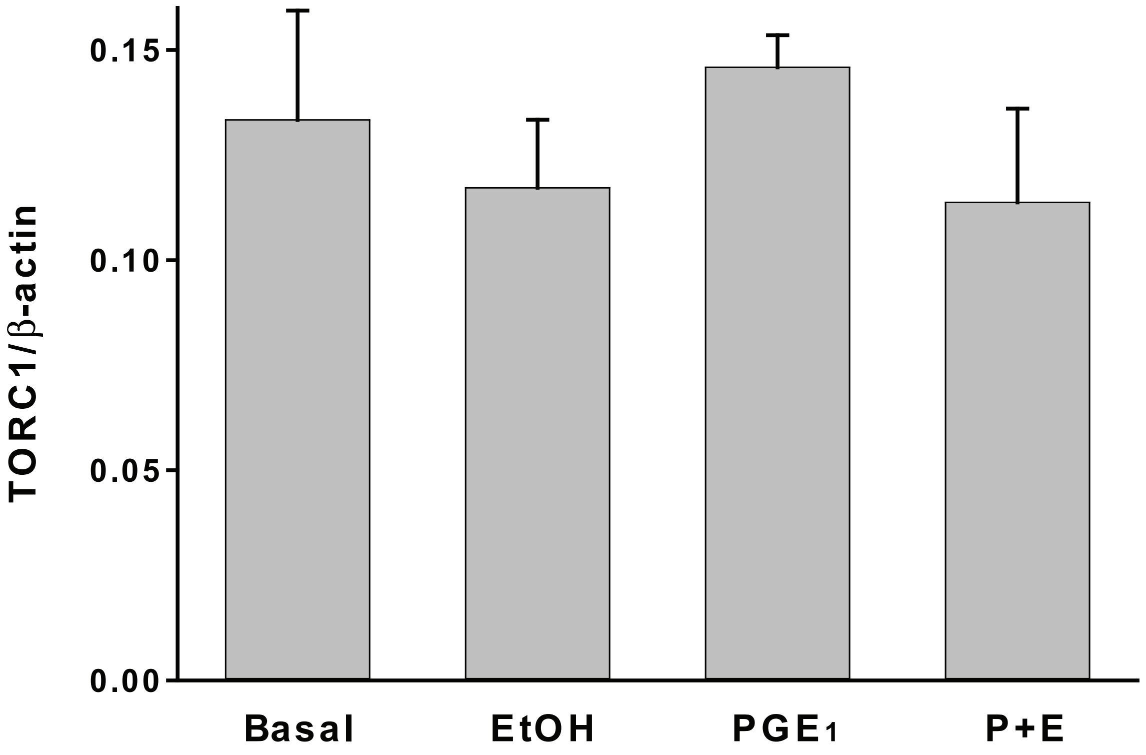

Supplementary Fig. S6. Immunodetection of TORC1. Changes in TORC1 were normalized using β-actin (n = 3). Changes in TORC1 in response to cAMP pathway activation or EtOH treatment are not significant (one-way ANOVA).
